# Body Exposure, its Forms of Delivery and Potentially Associated Working Mechanisms: How to Move the Field Forward

**DOI:** 10.32872/cpe.3813

**Published:** 2021-09-30

**Authors:** Andrea S. Hartmann, Eva Naumann, Silja Vocks, Jennifer Svaldi, Jessica Werthmann

**Affiliations:** 1 Institute of Psychology, Osnabrück University, Osnabrück, Germany; 2 Department of Psychology, Eberhard-Karls University Tübingen, Tübingen, Germany; 3 Institute of Psychology, Albert-Ludwig University Freiburg, Freiburg, Germany; Philipps-University of Marburg, Marburg, Germany

**Keywords:** body exposure, eating disorders, body image disturbance, working mechanisms, intervention

## Abstract

**Background:**

Body image disturbance (BID) is a hallmark feature of eating disorders (EDs) and has proven to be involved in their etiology and maintenance. Therefore, the targeting of BID in treatment is crucial, and has been incorporated in various treatment manuals. One of the most common techniques in the treatment of BID is body exposure (BE), the confrontation with one’s own body. BE has been found to be effective in individuals with EDs or high body dissatisfaction. However, BE is applied in a multitude of ways, most of which are based on one or a combination of the hypothesized underlying working mechanisms, with no differential effectiveness known so far.

**Method:**

The aim of this paper is to selectively review the main hypothesized working mechanisms of BE and their translation into therapeutic approaches.

**Results and Conclusion:**

Specifically, we underline that studies are needed to pinpoint the proposed mechanisms and to develop an empirically informed theoretical model of BE. We provide a framework for future studies in order to identify working mechanisms and increase effectiveness of BE.

## Highlights

- Body exposure (BE) is an effective intervention for body image disturbance in eating disorders.
- Ways of delivery vary depending on assumed underlying working mechanisms.
- Impact of attention focus, verbalization, therapist presence, and dosage should be investigated.
- Research on working mechanisms will improve BE and maximize results for specific patients.

Body image disturbance (BID) is a distinct risk factor for the development and maintenance of eating disorders (EDs), and potentially contributes to relapse after treatment (e.g., Glashouwer et al., 2019). Furthermore, targeting body dissatisfaction is associated with better overall treatment outcome (Wilson et al., 2002). Thus, the improvement of body image should be a key element of ED treatment, e.g. in the form of body exposure (BE), alongside the normalization of nutrition and eating behaviors. This paper aims to selectively review the theoretical rationales underlying potential working mechanisms of BE, the empirical evidence for these rationales, and the corresponding therapeutic application of BE. Another aim is to review future research ideas on mechanisms, BE delivery, and moderators of BE effects in order to foster clinicians’ use of BE as an effective intervention strategy.

## Efficacy of Body Exposure

A meta-analytical review indicated that BE is effective as stand-alone intervention for BID (Alleva et al., 2015). The analysis included 62 original studies on the effectiveness of stand-alone interventions to improve body image that had a control group, random allocation to conditions, and at least one pre- and posttest measure. Two interventions that can be broadly viewed as BE - namely exposure exercises and guided imagery exercises - showed significant intervention effects on body image. The meta-analysis further demonstrated that effects were stronger when targeting individuals with body concerns as compared to unselected groups (Alleva et al., 2015). In an extension of this finding, a more recent review (Griffen et al., 2018) focused on summarizing the effects of BE in distinct groups of individuals with various ED diagnoses separately and mixed, as well as individuals with obesity, body dysmorphic disorder, and non-clinical individuals. Their search yielded a total of 15 studies evaluating BE. For all participant groups, at least preliminary effectiveness of BE was shown. However, due to a scarcity of studies no differential effectiveness of various forms of BE could be determined (Griffen et al., 2018).

Notably, some individuals do not benefit from BE, as evidenced by findings that on certain measures, between-group effects are significant while group by time interaction effects are not (e.g., Delinsky & Wilson, 2006). Research and reports on symptom deterioration or treatment dropouts are rare. In a randomized controlled trial by Hildebrandt and colleagues (2012), self-injurious behaviors and subsequent study dropout occurred in the BE condition but not the control condition. In a study by Delinsky and Wilson (2006), the only dropouts occurred in the BE condition (without significant attrition differences between conditions), and the participants who dropped out also had higher depression scores at the outset. Accordingly, while BE might deteriorate symptoms in emotionally unstable patients, frequency of symptom deterioration or treatment discontinuation cannot be extrapolated from current data.

In sum, BE seems to be effective for the majority of patients. A common characteristic of BE procedures is a systematic examination of one’s own body by the patient – in a mirror or through recorded videos – over a varying number of sessions. However, different BE versions exist in which the specific BE approach varies in several aspects, and the (clinical) decision for the specific BE approach often relies on the hypothesized underlying working mechanism.

## Hypothesized Working Mechanisms: Theoretical Ideas on How BE Reduces Body Image Disturbance

The theoretical accounts of BE show distinct differences, resulting in a variety of specific intervention approaches. Here, we will briefly review four theoretical ideas that have previously been proposed. Moreover, where available, we present empirical evidence and the respective treatment implications. Of note, the field is only just beginning to develop a comprehensive understanding of how exposure might work, and an integrated model of these rationales is lacking. Thus, while in the following the theoretical ideas are discussed as discrete working mechanisms, it might very well be that they all work alongside each other or interact (Lass-Hennemann et al., 2018). Furthermore, there may also be a general working mechanism, e.g., the generally structured preoccupation with one’s body without avoidance or safety behaviors.

First, a hypothesis derived from exposure research in anxiety disorders posits that habituation to negative emotion and distress on psychological and biophysiological processing levels is responsible for the positive effects of BE. From a theoretical perspective, repeated and prolonged exposure to the conditioned stimulus ‘‘seeing one’s own body’’ (CS) is assumed to induce decreases in the conditioned negative reaction (CR) by preventing negative reinforcement, e.g., avoidance (Benito et al., 2018; Craske et al., 2014). Indeed, there is evidence for a reduction of self-reported negative affect between and within exposure sessions (e.g., Trentowska et al., 2017). While these findings are supported by some studies assessing physiological parameters (e.g. emotional arousal measured by means of voice stress analysis; Baur et al., 2020), other findings, e.g. from studies assessing heart rate as a physiological measure of change in distress during BE, are more ambiguous (Trentowska et al., 2017; Vocks et al., 2007). One reason for this inconsistency might be that BE elicits a multitude of emotions in individuals with BID (e.g., Naumann et al., 2013). For instance, in individuals with EDs, disgust has been shown to play a more important role than anxiety (e.g., von Spreckelsen et al., 2018). Moreover, disgust seems more resistant to psychological and physiological habituation processes in other disorders (Olatunji et al., 2009), and is influenced more likely by counterconditioning (e.g., Engelhard et al., 2014). Recently, potential working mechanisms of exposure (in anxiety research) have been overhauled by the so-called inhibitory learning approach. Accordingly, the working mechanism of exposure lies in the development and strengthening of nonthreat associations in memory during exposure (e.g., Craske et al., 2008; Foa & McLean, 2016).

Thus, within an exposure framework of BE, three potential working mechanisms have been suggested: habituation, counterconditioning, and inhibitory learning. While all three approaches are based on an exposure rationale, each offers a distinct and differential therapeutic application of BE in a clinical context. Treatment manuals postulating habituation as a working mechanism recommend that patients mainly focus on their negatively valenced body parts over an extended period of time in order to activate negative affect, which consequently can be reduced (Vocks et al., 2018). Treatment manuals based on the counterconditioning mechanisms should aim to change the unwanted reaction (negative affect) when confronted with the stimulus (body). Thus, they might suggest to rather focus on positively valenced body parts, coupled with an instruction to do something positive for/with one’s body (e.g., use body lotion) or, to focus on negatively valenced body parts while instructing to elicit positive thoughts about the body and/or remember what the body already has achieved (e.g, Vocks et al., 2018). And lastly, treatment manuals using inhibitory learning as a rationale would aim to use as many different exposure exercises as possible in order to maximize the possibilities to create nonthreat associations.

Another theoretical rationale of BE is based on the idea of attention bias modification. The hypothesis was derived from data demonstrating a negative attentional bias to subjectively unattractive body parts when confronted with one’s own body in individuals with EDs (e.g., Bauer et al., 2017). It was hypothesized that a change in this dysfunctional attention pattern might alter the associated negative affect. Some studies have demonstrated that a focus on positively valenced body parts leads to an improvement on measures of body image (Glashouwer et al., 2016; Krohmer et al., authors’ unpublished data; Smeets et al., 2011), and some (Krohmer et al., authors’ unpublished data) but not all (Glashouwer et al., 2016) have reported a concurrent change in attention patterns. However, one study did not find differential effects between a negative and a positive focus condition on body dissatisfaction, body-related checking, body concerns, and negative mood from pre- to post-BE (e.g., Jansen et al., 2016). This contradicts the idea of attention bias modification as the only working mechanism of BE. Following this rationale, corresponding therapeutic BE approaches asked patients to focus on positively valenced body parts only (Jansen et al., 2016; Vocks et al., 2018) or to state their emotional connotations of the respective body parts while distributing their attention evenly (Svaldi & Tuschen-Caffier, 2018).

A third theoretical rationale of BE is based on the hypothesis of reduction of body perception distortion in individuals with EDs. Most individuals with EDs overestimate the dimensions of their own body (e.g., Mohr et al., 2016; Volpe et al., 2018). Furthermore, there is some (Norris, 1984), but also contrasting (Lewer et al., 2017; Vocks et al., 2007) evidence that distorted body perception might change over the course of BE. More recently, a systematic review suggested that the construct of distorted perception may be misleading as the distortion may rather stem from a dysfunctional cognitive-evaluative component of body image than from perceptual deficits (Mölbert et al., 2017). Following this rationale, one would advise an even distribution pattern and the use of non-judgmental language (Hildebrandt et al., 2012) during BE.

A fourth theoretical rationale suggests that central dysfunctional cognitions (e.g., interpretation and memory biases, e.g., Korn et al., 2020) of BID are changed through (implicit) cognitive restructuring in the course of BE. Such cognitive restructuring can be achieved by inducing cognitive dissonance (e.g., between the dysfunctional belief “My stomach looks fat” and the behavior of describing the stomach neutrally), which may in turn reduce body-related negative schemata (Williamson et al., 2004). In addition to the above-mentioned induction of cognitive dissonance and cognitive restructuring, therapeutic approaches of BE derived from this hypothesis instruct patients to either focus on positively valenced body parts or to focus on all body parts evenly, while describing their body positively or neutrally (i.e., with the therapist present; e.g., Jansen et al., 2016; Klimek et al., 2016; Luethcke et al., 2011).

All of these aspects are noteworthy, as BE seems, in general, a promising tool to address body image disturbances in clinical and non-clinical populations (Alleva et al., 2015), even though with only small effect sizes as a stand-alone technique in the latter. Accordingly, there is a need to refine the theoretical rationale as well as (experimental) research on working mechanisms in order to improve the technique and potentially individualize it in the future to maximize outcome in specific patients.

## Suggested Foci in Future Research

It is important for future research to focus on factors that determine its positive effects. In the following, we describe variables that require systematic examination.

### Where Should One Look During BE?

As briefly reviewed above, depending on the theoretical rationale, BE approaches differ in whether patients are instructed to focus selectively on positively or negatively valenced body parts, or evenly on all body parts. Given that these foci might elicit emotions that may or may not be necessary to reach the intervention goal, it is essential to understand individual needs and differences. In one study, interventions with a focus on exclusively positive or negative body parts successfully reduced body dissatisfaction, body-related checking, body concerns, and negative mood in women with high levels of body dissatisfaction (Jansen et al., 2016). Moreover, the negative focus condition yielded a stronger decrease in body-related avoidance behavior over the follow-up period. For comparison studies, we propose to consider another effective form of BE, which comprises instructions to focus on all body parts from head to toe, successively, in order to correct distorted body perception and alter viewing patterns. Furthermore, we suggest testing a form in which body parts are clustered by their indication of weight gain or status (e.g., thighs, bottom, stomach vs. knees, ankles, forearms), instead of by their subjective valence. This might be of particular interest if the hypothesized working mechanism is dissolution of the conditioned association, as it allows for exposure to the most fear-inducing body parts, given that fear of weight gain is a central concept of individuals with EDs (e.g., Rodgers et al., 2018).

### How Should Verbalization Be Instructed During BE?

Another large difference between previous studies lies in the type of body-related descriptions provided by participants, i.e. whether they purely describe their body, or the associated emotions and cognitions, or both. While a negatively toned description might strengthen the experience of BE (in the sense of a stronger habituation effect), subsequently leading to a more effective dissolution of negative body-related affect, a mainly positive or neutral, non-judgmental description might strengthen the decrease in negative affect by correcting distorted perception, thus altering dysfunctional attention processes or cognitive dissonance processes (rather like inhibitory learning). So far, only two studies have compared different forms of instructed verbalizations. In the first study, the authors compared two neutral versions of BE to a cognitive dissonance version in which participants were instructed to describe body parts using positive verbalizations. While all three forms led to improvements on measures of ED and body image, only the cognitive dissonance version of BE yielded an increase in body satisfaction (Luethcke et al., 2011). In the second study, a positive and a negative full-body verbalization condition were compared in healthy individuals. Both interventions yielded improvements in emotional arousal and body satisfaction between sessions. However, within sessions, the negative but not the positive verbalization condition led to a decrease in positive affect and body satisfaction and an increase in negative affect (Tanck et al., authors’ unpublished data). To further disentangle different forms of verbalisation, we propose to compare a neutral description of what patients see, and a description of positive or negative aspects of each body part in future studies. Thereby, while manipulating the form of verbalization, the attentional focus should be controlled (e.g., by asking patients to describe every part of their body from head to toe).

### Is a Therapist Needed in BE?

To the best of our knowledge, there are no studies comparing BE with and without a therapist present. Such investigations would be highly relevant, as the presence of a therapist could impact the effectiveness of the intervention, particularly when considering cognitive dissonance as a working mechanism. Comparative studies have looked at differences in the effectiveness of guided vs. unguided BE (Díaz-Ferrer et al., 2015; Díaz‐Ferrer et al., 2017; Moreno-Domínguez et al., 2012). For example, women with body dissatisfaction and subclinical EDs underwent either an *unguided* version, in which they freely explored self-chosen body parts and were instructed to verbalize associated emotions and cognitions, or a *guided* version, in which they focused on all body parts and had to describe them using neutral words. Both conditions were found to be effective in reducing BID, with a slight superiority of the unguided condition. However, heart rate and skin conductance observed within sessions indicated that the two techniques might act through different mechanisms (Díaz‐Ferrer et al., 2017), with a stronger increase in both indicators in the unguided condition. Notably, the conditions in the comparison studies varied not only with respect to therapists’ active guidance during BE, but also regarding the body parts which were focused on and the way in which body parts were described. Thus, in order to understand the impact of therapist presence and guidance during BE, future research should compare guided and unguided versions of BE while controlling for focus and type of verbalization.

### How Much BE Is Needed?

The ideal intensity of BE remains unclear. On the one hand, intensity can be captured as frequency of sessions. In anxiety disorder research, the frequency of exposure is assumed to be a major factor in treatment effectiveness (Wolitzky-Taylor et al., 2008). In EDs, several findings highlight that therapeutic effects might occur mostly between rather than within sessions (e.g., Hilbert et al., 2002). Thus, multiple sessions are necessary, which is further underlined by the finding that short-term exposure leads to an activation and deterioration of body satisfaction and negative affect (Veale et al., 2016). Findings from studies investigating the effects of different numbers of sessions are important, because they may, for instance, allay clinicians’ fears of overwhelming the patient when delivering multiple BE sessions.

On the other hand, intensity can also be captured as duration of single sessions, thus the length of a BE therapy session, BE sessions over a whole day, or exposure until a reduction in anxiety to a certain predefined extent is realized. In intensive exposure (“flooding”), aversive stimuli are presented at the highest level of intensity, while gradual exposure follows a stepwise approach starting at a low level of intensity. Previous research in the area of obsessive-compulsive disorder suggests that intensive exposure might lead to a stronger short-term reduction of anxiety symptoms. By contrast, gradual exposure might be more helpful for reducing emotions that habituate more slowly, such as disgust (Olatunji et al., 2009). More recent studies in the area of anxiety disorders advocate for variability in the exposure hierarchy in order to maximize inhibitory learning (e.g., Knowles & Olatunji, 2019). Future research should test whether variations in intensity impact BE effects on BID. Besides frequency and duration of sessions, potentially relevant moderating variables in the context of intensity of BE may relate to the setting (e.g., mirror size, light, distance to mirror) or clothing (everyday vs. tight clothes vs. underwear).

### Who Benefits or Does not Benefit From BE?

Evidence of differential effectiveness of BE in specific groups is limited by the low diversity of the groups researched so far. Men have been overlooked in body image research, including BE interventions (Burlew & Shurts, 2013), and BE in individuals with comorbidities remains to be investigated. Additionally, as Alleva et al. (2015) highlighted, individuals of middle to older age have also been neglected in past BE research.

Furthermore, for body dysmorphic disorder, another mental illness with the core symptom of BID, BE (mirror retraining), also represents an essential part of the CBT protocol (e.g., Wilhelm et al., 2013). However, to date, no study has examined the effectiveness of this technique detached from the overall CBT treatment. Further research into the effectiveness of BE in mental disorders potentially associated with BID, namely borderline personality disorder, posttraumatic stress disorder, or social anxiety disorder (Dyer et al., 2013; Dyer et al., 2015) is also lacking.

Lastly, a comprehensive evaluation of BE effectiveness should also include the systematic assessment of side effects, adverse events, or predictors of non-responders, and a subsequent trade-off between positive effects and negative aspects for single patient groups. As looking at oneself in a mirror can lead to significant distress and a worsening of negative affect (Veale et al., 2016; Walker et al., 2012; Windheim et al., 2011), BE might destabilize some patients. Eventually, extending research to subgroups will help to formulate diagnosis- and patient group-specific treatment guidelines, which will move us closer to establishing individualized evidence-based treatments.

### What Might Further Influence the Efficacy of BE?

Several potential moderators may be worthy of further investigation, because they may have confounded previous research results. Moderating factors may also influence practitioner’s decision to implement BE. Given the scarcity of previous research, we are not able to quantify the impact of, for example, current weight, gender- and weight-match between patient and therapist, current status of treatment, chronicity of symptoms, level of habitual checking and avoidance, and the delivery in groups vs. alone on the effectiveness of BE. We suggest that all of these factors should be assessed in future studies to provide information regarding their impact on BE effects and on clinician’s decision to implement BE.

### Tools for Evaluating BE Mechanisms and Efficacy

Past studies varied regarding outcome and process variables. To understand the differential effectiveness of BE on various levels of experience, a comprehensive set of process and outcome measures needs to be considered. First, we suggest that different facets of body image should be assessed in order to capture processes and outcomes on all levels of BID (i.e. perceptual, cognitive-affective, and behavioral). Second, we advocate for the adoption of a multi-method approach encompassing self- and expert-report measures, as well as objective measures in order to elucidate mechanisms of BE on as many processing levels as possible. The former might include self- and external report measures on body dissatisfaction and disorder-specific symptomatology. The latter might consist of psychobiological indicators of emotional activation indexing fear- and anxiety-related differences in the autonomic nervous system, e.g., such as fear-potentiated startle and heart rate, but also indices of attention allocation and information processing as well as the very recent approach of vocal arousal.

## Conclusion

Despite findings regarding the effectiveness of BE in intervention studies, it is still largely unknown which version works best for whom. Thus, first, lab-based experimental studies need to be conducted to isolate the effect of potential working mechanisms and test their impact within the different proposed forms of BE on BID outcomes (Glashouwer et al., 2020). Current studies from our workgroups target this research gaps by setting out to differentiate attention foci and verbalization forms measuring self-reported, peripherphysiological, and eye-tracking outcomes. Findings from these and other studies can then inform theory-based and empirically based models on key processes, and can advance refined etiological models of BID. In the future, interventions based on these models can then be tested in larger randomized controlled trials including additional analyses of moderators to identify which specific BE procedure is maximally successful (or unsuccessful) for a specific patient subsample. Of further relevance, research needs to prove that the positive effects of BE outweigh the fact that this technique can be strenuous for patients, as they are confronted with the very thing they fear the most.

## Data Availability

Data sharing is not applicable to this article as no new data were created and analyzed in this study.
